# CO-PRODUCING A BOARDGAME TO LEARN AND ENGAGE ABOUT DEMENTIA INEQUALITIES: THE DEMENTIA INEQUALITIES GAME

**DOI:** 10.1093/geroni/igad104.3246

**Published:** 2023-12-21

**Authors:** Clarissa Giebel, Kerry Hanna, Hilary Tetlow, Mark Gabbay, Jacqui Cannon

**Affiliations:** University of Liverpool, Liverpool, England, United Kingdom; University of Liverpool, Liverpool, England, United Kingdom; SURF Liverpool, Liverpool, England, United Kingdom; University of Liverpool, Liverpool, England, United Kingdom; Lewy Body Society, Wigan, England, United Kingdom

## Abstract

Receiving a diagnosis and accessing care after a diagnosis of dementia, both for the person and their carer, are fraught with inequalities. Finding different ways to address these inequalities is important. The aim of this public engagement activity was to co-produce a boardgame about dementia inequalities to facilitate learning, dialogue and educate about different barriers, and facilitators, to diagnosis and care. Two virtual and two face-to-face workshops with people with dementia, unpaid carers, health and social care professionals, and Third Sector representatives were held between October 2022 and June 2023. The virtual workshops were split into discussions of inequalities and how a boardgame may feature inequalities. The first face-to-face workshop was split into the same activities, aided by outcomes from workshops 1 and 2. Workshop 4 attendees tested the prototype. Attendees were reimbursed for their time. Nine brief remote interviews with attendees were conducted to evaluate their experiences in the co-production of the boardgame. Forty stakeholders attended four workshops. Workshops provided step-by-step thoughts on how the game could be designed or modified. The final game, prototype tested in Workshop 4, consists of a one-sided, two-half board depicting the pre-diagnosis process (left half) and post-diagnosis process (right half). Interviews with nine attendees reported on their reasons for attending the workshops, experiences of being involved, and the game design and purpose. The game can be used to improve knowledge about dementia inequalities for health and social care professionals, carers, people living with dementia, decision makers, and the general public.

